# The Efficacy of Educational Interventions in Improving School Teachers’ Knowledge of Attention Deficit Hyperactivity Disorder

**DOI:** 10.7759/cureus.44509

**Published:** 2023-09-01

**Authors:** Ebtihal E Eltyeb, Gassem A Gohal, Nirmin H Alhazmi, Sulaiman Hamdi, Layla H Al khairat, Nawaf A Shutayfi, Alaa H Al-Khairat, Halimah A Sumayli, Taher A Someli, Sharifah A Someli

**Affiliations:** 1 Department of Pediatrics, College of Medicine, Jazan University, Jazan, SAU; 2 Faculty of Medicine, College of Medicine, Jazan University, Jazan, SAU

**Keywords:** attention deficit hyperactivity disorder (adhd), teachers, saudi, knowledge, educational intervention

## Abstract

Background: Well-trained primary school teachers should be competent in recognizing attention deficit hyperactivity disorder (ADHD) in students and be able to assist in providing care and support.

Objectives: This study intends to assess primary school teachers' knowledge of ADHD and evaluate the effectiveness of a short-term educational intervention.

Methods: A quasi-experimental quantitative study was conducted among primary school teachers in Jazan, Saudi Arabia. A two-hour educational intervention was designed and run in six schools using a specific knowledge rating scale before and after the application of the intervention.

Results: A total of 150 primary school teachers were included in this study, of which 64% were males, 51.3% were in the age group of 40-49 years, and 28% had teaching experience of more than 20 years. Regarding ADHD knowledge, the pre-intervention knowledge of the general criteria, symptoms and diagnosis, and treatment was considered adequate in 3.3%, 16.7%, and 2.7% of the participants, respectively, which improved post-intervention to 22%, 54.7%, and 19.3%, respectively. There was a significant association between the pre-intervention knowledge of the general criteria and the gender and between the knowledge of symptoms and diagnosis and the attendance of ADHD workshop by the participants. Also, there was a significant association between the participants' age, residence experience, attendance of ADHD workshops, and the general criteria and treatment domains.

Conclusions: Most primary school teachers in Jazan have insufficient knowledge of ADHD. Using a knowledge improvement intervention can substantially improve the teachers’ knowledge. Therefore, it is necessary to incorporate thoughtful knowledge improvement programs into the educational curricula for teachers.

## Introduction

The most common neuro-behavioral disease affecting children is attention deficit hyperactivity disorder (ADHD), which affects 3.4-7.2% of children globally and 2.7-16.4% of children in Saudi Arabia [1-3]. The two main characteristics that distinguish ADHD in children are inattentiveness and hyperactivity/impulsivity in at least two settings, usually home and school. Moreover, the disorder affects social, intellectual, and occupational functioning in various settings. In primary school, such children tend to have trouble paying attention in class, have poor capacity for organization, have difficulty interacting with others, and possibly have problems being autonomous. The diagnosis of ADHD in children requires unique rating scales and should be done at specific ages, with symptoms that must have been present for at least six months [4,5]. There are two modes of ADHD treatment: psychosocial and pharmaceutical stimulant medications.

Because most children spend more time in school and interact with teachers than with their parents or healthcare professionals, primary school teachers have taken responsibility for assessing children's academic and behavioral difficulties and providing immediate assistance shortly after early detection. Interventions for ADHD teacher training have been designed to increase teachers' understanding of the disorder, prepare them to foster a supportive learning environment, and help them develop problem-solving tactics [6]. Recent approaches to therapy require social interaction, including school-based activities, which necessitate skilled teaching staff [7].

There is a reasonable effort in Saudi Arabia to diagnose, manage, and raise awareness of ADHD. For example, the Saudi Ministry of Health started a national School-Based Screening Program (NSBSP) to examine children's many health conditions, including ADHD [8]. Moreover, Saudi Arabia approved the recently disseminated guidelines for the National Institute for Health and Care Excellence (NICE) ADHD diagnosis and management [9]. Furthermore, many projects are arranged by the Saudi ADHD Society to train educators, organize summer camps, and develop support groups for the families of the affected children [10]. Despite that, studies in Saudi Arabia have demonstrated that there is still a lack of knowledge, perception, and attitudes regarding ADHD among the population generally and teachers in particular [11-14]. Therefore*,* this study aims to assess the knowledge of ADHD among primary school teachers in the Jazan region of Saudi Arabia and evaluate the impact of the socio-demographic variables on the knowledge before and after interventional educational lectures.

## Materials and methods

Study design and participants

This quantitative quasi-experimental study was conducted in the city of Jazan, the capital of the Jazan region in the Kingdom of Saudi Arabia, between February and June 2023. The study was conducted among primary school teachers, both male and female, in six schools. The study was approved by the Standing Committee for Scientific Research, Jazan University (HAPO-10-Z-001) (approval number: REC-REC-44/06/476, dated January 5, 2023).

This study included all teachers in the selected primary schools who agreed to participate, and excluded the teachers who did not match the inclusion criteria, refused to participate, or did not complete the questionnaire. The minimum sample size was 150 participants, calculated by an online sample size calculator [15], utilizing a 95% confidence interval, a population size of 8732 (according to the available data from the Ministry of Education in Jazan) [16], a margin of error of 5%, and a response of distribution of 10%. Then, a convenient sample was taken from the selected schools.

Data collection tool and the collection procedure

Data collection was done with the help of a questionnaire that had questions on demographic information (age, gender, residence, educational level, years of experience, and if the participant has attended any ADHD workshop before), previous experience and knowledge regarding ADHD; the last section assessed the participants' knowledge using the Knowledge of Attention Deficit Disorders Scale (KADDS) that was created by Sciutto and colleagues in 2000 to assess knowledge gaps and common misconceptions among parents of children with ADHD, educators of those children, and other clinical and educational professionals [17]. The 36 statements on ADHD in the KADDS, with responses graded as 'incorrect,' 'correct,' and 'do not know,' are distributed into three domains: ADHD symptoms and diagnosis domain (nine questions), ADHD treatment domain (12 questions), and General features of the illness domain (15 questions) (See Appendix). The KADDS questionnaire's English version was translated into Arabic under the direction of a native-speaker professional panel with experience in translation. Next, the authors conducted a pilot study among 15 participants to ensure readability, length, and comprehension of the questionnaire items, for which participants were excluded from the latter sample size. The questionnaire's internal consistency was estimated based on Cronbach's alpha and yielded a sufficient value of 0.888. For internal consistency, the varimax rotation method was employed, and the normality of data was tested using the Kolmogorov-Smirnov test and the Shapiro-Wilk test. Kaiser-Meyer-Olkin measure was used to determine sampling adequacy and was 0.773 (77.3%). In addition, Bartlett's Test of Sphericity was done and was statistically significant (p <0.001).

The participants filled out an informed consent that demonstrated the purpose and the method of the study; then, they received the questionnaire to be filled out before the educational intervention. Because of the time constraints, the educational interventional program was prepared as a two-hour lecture that contained the basic knowledge of ADHD regarding general features, symptoms, warning signs, diagnosis, and treatment, which enabled the participants to answer all the given questions in the KADDS questionnaire. The authors prepared the lecture in an uncomplicated, understandable PowerPoint (Microsoft Corporation, Redmond, Washington, United States) format with visual illustrations to attract the participants. The participants were permitted to ask questions after the demonstration to ensure their understanding of the concepts in the lecture. Then, the same questionnaire was used to assess teachers' knowledge of ADHD immediately following the interventional educational lecture was concluded.

Knowledge Score

Each correct answer in the questionnaire was one mark; wrong answers and "do not know" got zero marks. This calculation gave a score of 0-36 and then converted to percentages. The score was not normally distributed, so we calculated 25% and 75% of the data, and the knowledge was categorized into Poor (< 25%), Fair (25-75%), and Adequate (> 75%) [18].

Data presentation and statistical analysis

IBM SPSS Statistics for Windows, Version 23.0 (Released 2015; IBM Corp., Armonk, New York, United States) was used for data analysis. Initially, all information gathered via questionnaire was then coded into variables. Frequencies were used to give a general overview of the data. Descriptive and inferential statistics involving the Pearson Chi-square test, one-way ANOVA, Fisher's exact test, and the Mc-Nemar-Bowker test were used to present the results. A p-value of less than 0.05 was considered statistically significant.

## Results

In total, 150 primary school teachers were included in this study. The participants' characteristics are shown in Table 1. Regarding age groups, participants in the 40-49 age groups comprised the highest percentage (51.3%); 67.3% resided around Jazan, 64% were males, and 28% of the participants had long-term teaching experience that extended more than 20 years. Only five participants (3.3%) had post-graduate education, and only 57 (38%) had attended ADHD workshops. 

**Table 1:** Demographic Characteristics of the Participants (N =150) ADHD: attention deficit hyperactivity disorder

Demographic variables	Number	Percent
Age Group		
Less than 30 years	5	3.3%
30-39 years	50	33.3%
40-49 years	77	51.3%
50 years and more	18	12%
Years of Experience		
Less than five years	7	4.7%
6-10 years	37	24.7%
11-15 years	29	19.3%
16-20 years	35	23.3%
More than 20 years	42	28%
Gender		
Male	96	64%
Female	54	36%
Residence		
Inside Jazan city	49	32.7%
Around Jazan city	101	67.3%
Educational Level		
Diploma	37	24.7%
Bachelor's degree	108	72%
Postgraduate degree	5	3.3%
Attended ADHD Workshops		
Yes	57	38%
No	93	62%

Regarding ADHD knowledge, Mc-Nemar's test for correlated proportions was applied with matched pre- and post-intervention pairs in the three domains. The pre-intervention knowledge regarding the general criteria domain, symptoms and diagnosis domain, and treatment domain was considered adequate (> 75%) in 3.3%, 16.7%, and 2.7% of the participants, respectively, which improved post intervention to 22%, 54.7%, and 19.3%, respectively, in the three domains. Also, the poor ADHD knowledge (< 25%) revealed a statistically significant improvement post intervention among the participants in the three domains (p = 0.000), as shown in Table 2. 

**Table 2:** Comparison Between Pre-Intervention and Post-Intervention ADHD Knowledge in the Participants ** P- value is significant < 0.05 ADHD: attention deficit hyperactivity disorder

		Post-Intervention Knowledge Regarding General Criteria			Total	p-value
Pre-knowledge regarding general criteria		Poor	Fair	Adequate		
Poor		51 (46.8%)	32 (29.4%)	26 (23.9%)	109 (72.7%)	0.000**
Fair		7 (19.4%)	23 (63.9%)	6 (16.7%)	36 (24%)	
Adequate		1 (20%)	3 (60%)	1 (20%)	5 (3.3%)	
Total		59 (39.3%	58 (38.7%)	33 (22%)	150 (100%)	
	Post-Intervention Knowledge Regarding Symptoms and Diagnosis					
		Poor	Fair	Adequate		
Poor		2 (3.4%)	25 (43.1%)	31 (53.4%)	58 (38.7%)	0.000**
Fair		4 (6%)	26 (38.8%)	37 (55.2%)	67 (44.7%)	
Adequate		2 (8%)	9 (36%)	14 (56%)	25 (16.7%)	
Total		8 (5.3%)	60 (40%)	82 (54.7%)	150 (100%)	
		Post-Intervention Knowledge Regarding Treatment				
		Poor	Fair	Adequate		0.000**
Poor		33 (32.7%)	48 (47.5%)	20 (19.8%)	101 (67.3%)	
Fair		10 (22.2%)	27 (60%)	8 (17.8%)	45 (30%)	
Adequate		1 (25%)	2 (50%)	1 (25%)	4 (2.7%)	
Total		44 (29.3%)	77 (51.3%)	29 (19.3%)	150 (100%)	

One-way ANOVA was used to assess if there were any statistically significant differences between the means of the three domains and the demographic variables of the participants. As shown in Table 3, there is a significant association between the pre-intervention knowledge of the general criteria domain and the gender and between the symptoms and diagnosis domain and the attendance of ADHD workshop by the participants. Moreover, Table 4 showed a significant association between the participants' age, residence experience, attendance of ADHD workshops, and the general criteria and treatment domains.

**Table 3:** One-way ANOVA Measuring the Pre-Intervention Knowledge Domains and the Different Demographic Variables (N=150) *p-value is significant < 0.05 SD: standard deviation; SE: standard error; ADHD: attention deficit hyperactivity disorder

Variables		Number	General criteria (%)				Symptoms and diagnosis (%)				Treatment (%)			
			Mean	SD	SE	p-value	Mean	SD	SE	p-value	Mean	SD	SE	p-value
Age groups	< 30 years	5	47.8	10.986	4.913	0.145	51	15.281	6.834	0.222	36.6	12.661	5.662	0.948
	30-39 years	50	43.46	17.058	2.412		52.92	19.099	2.701		38.8	15.431	2.182	
	40-49 years	77	37.78	15.397	1.755		57.21	18.495	2.108		37.12	17.358	1.978	
	≥50 years	18	42.94	15.307	3.608		62.44	13.008	3.066		37.94	11.522	2.716	
Gender	Male	96	44.01	15.315	1.563	0.001*	55.84	19.096	1.949	0.750	41.29	15.303	1.562	0.0002*
	Female	54	34.61	15.533	2.114		56.83	16.517	2.248		31.48	15.038	2.046	
Residence	Inside Jazan City	49	44.08	17.195	2.456	0.065	57.63	17.763	2.538	0.503	40.8	16.758	2.394	0.103
	Around Jazan City	101	38.95	15.186	1.511		55.5	18.395	1.83		36.29	15.3	1.522	
Educational level	Diploma	37	40.97	17.241	2.834	0.953	54.05	17.461	2.871	0.328	38.97	15.062	2.476	0.808
	Bachelor's degree	108	40.6	15.653	1.506		56.44	18.507	1.781		37.24	16.293	1.568	
	Postgraduate degree	5	38.6	17.155	7.672		66.8	13.882	6.208		40	14.816	6.626	
Years of experience	< 5 years	7	47.43	15.109	5.711	0.219	47.57	16.92	6.395	0.064	34.43	16.999	6.425	0.121
	6-10 years	37	39.62	18.447	3.033		55.95	19.214	3.159		38.76	16.669	2.74	
	11-15 years	29	42.1	13.707	2.545		49.38	19.6	3.64		33.9	14.113	2.621	
	16-20 years	35	40.74	14.873	2.514		58.46	18.192	3.075		43.29	17.875	3.021	
	> 20 years	42	39.26	16.54	2.552		60.69	14.995	2.314		35.5	13.572	2.094	
Attended ADHD Workshop	Yes	57	42.68	14.194	1.88	0.747	61.11	15.971	2.115	0.01*	38.82	13.621	1.804	0.522
	No	93	39.37	16.956	1.758		53.19	18.835	1.953		37.11	17.152	1.779	

**Table 4:** One-way ANOVA Measuring the Post-Intervention Knowledge Domains and the Different Demographic Variables (N=150) *p-value is significant < 0.05 SD: standard deviation; SE: standard error; ADHD: attention deficit hyperactivity disorder

Variables		Number	General criteria (%)				Symptoms and diagnosis (%)				Treatment (%)			
			Mean	SD	SE	p-value	Mean	SD	SE	p-value	Mean	SD	SE	p-value
Age groups	< 30 years	5	68	8.631	3.86	0.01*	67	7.778	3.479	0.877	60.2	13.664	6.111	0.035*
	30-39 years	50	59.42	14.171	2.004		71.78	11.552	1.634		60.16	16.765	2.371	
	40-49 years	77	50.22	19.851	2.262		70.94	14.784	1.685		50.66	20.367	2.321	
	≥50 years	18	50.22	14.49	3.415		71.89	10.819	2.55		51.39	16.245	3.829	
Gender	Male	96	53.83	17.695	1.806	0.966	71.42	12.882	1.315	0.788	53.21	18.805	1.919	0.379
	Female	54	53.96	18.281	2.488		70.81	13.544	1.843		56.06	19.29	2.625	
Residence	Inside Jazan City	49	58.73	16.053	2.293	0.02*	74.18	6.939	0.991	0.051	59.06	17.176	2.454	0.029*
	Around Jazan City	101	51.52	18.269	1.818		69.75	15.014	1.494		51.89	19.426	1.933	
Educational level	Diploma	37	56	16.092	2.645	0.498	73.84	11.405	1.875	0.032*	53.35	19.385	3.187	0.376
	Bachelor's degree	108	53.49	17.71	1.704		70.92	12.247	1.178		55.05	18.472	1.777	
	Postgraduate degree	5	46.6	32.129	14.369		57.8	30.02	13.425		43.2	26.678	11.931	
Years of experience	< 5 years	7	62.71	10.128	3.828	0.01*	71.71	8.655	3.271	0.970	60.71	14.874	5.622	0.013*
	6-10 years	37	61.08	17.864	2.937		71.7	11.645	1.914		61.97	16.36	2.69	
	11-15 years	29	55.41	19.657	3.65		69.9	14.967	2.779		55.76	22.419	4.163	
	16-20 years	35	49.43	18.6	3.144		70.71	15.518	2.623		50.2	21.042	3.557	
	> 20 years	42	48.71	14.336	2.212		71.98	11.703	1.806		48.64	14.853	2.292	
Attend ADHD Workshop	Yes	57	49.07	17.02	2.254	0.01*	69.65	13.53	1.792	0.257	49.25	18.223	2.414	0.011*
	No	93	56.83	17.788	1.845		72.15	12.781	1.325		57.29	18.856	1.955	

The Fisher's exact test and Pearson chi-square were used to assess whether there is a statistically significant relationship between demographic variables and the knowledge of ADHD. Age group, gender, educational level, and years of experience were significantly related to ADHD knowledge domains, as shown in Table 5.

**Table 5:** Relationship Between Participants’ ADHD Knowledge Domains and the Demographic Variables (N=150) * Fisher's Exact Test; **Pearson Chi-Square Test, p-value is significant < 0.05 ADHD: attention deficit hyperactivity disorder

Variables		Pre-intervention (general criteria)	Post-intervention (general criteria)	Pre-intervention (symptoms and diagnosis)	Post-intervention (symptoms and diagnosis)	Pre-intervention (treatment)	Post-intervention (treatment)
		P-values					
Age groups	< 30 years	0.135*	0.01*	0.836*	0.581*	0.967*	0.079*
	30-39 years						
	40-49 years						
	≥50 years						
Gender	Male	0.056*	0.950**	0.894**	0.958*	0.01*	0.349**
	Female						
Residence	Inside Jazan City	0.084*	0.142**	0.653**	0.0967**	0.197*	0.113*
	Around Jazan City						
Educational level	Diploma	0.627*	0.657*	0.465*	0.024*	0.156*	0.792*
	Bachelor's degree						
	Postgraduate degree						
Years of experience	< 5 years	0.293*	0.004*	0.539*	0.191*	0.042*	0.001*
	6-10 years						
	11-15 years						
	16-20 years						
	> 20 years						
Attend ADHD Workshop	Yes	0.892*	0.083**	0.189**	0.374*	0.551*	0.087*
	No						

## Discussion

This study aimed to assess the knowledge of primary school teachers working in Jazan, Saudi Arabia, before and after implementing an educational intervention on ADHD. The common incorrect KADDS items that showed poor knowledge were the estimates suggesting that ADHD occurs in approximately 15% of school-age children (42%), the possibility for an adult to be diagnosed with ADHD (40.7%), and the fact that ADHD children are more compliant with their fathers than with their mothers (37.3%). Also, 32.7% of the participants thought that ADHD is primarily the result of ineffective parenting, and 30.7% thought that treatment for ADHD that focuses mainly on punishment is the most effective in reducing the symptoms of ADHD.

Before the intervention, most teachers (73.3%) showed adequate knowledge that symptoms of ADHD must manifest before the age of seven to be diagnosed. Additionally, two-thirds (66.7%) of the teachers knew that children who frequently fidget or seem restless in two or more settings (such as at home and school) must exhibit relevant symptoms of ADHD. These outcomes aligned with research conducted in the cities of Madinah and Makkah regarding primary teacher knowledge and showed similar teachers' concepts [18,19].

Similar to previous Saudi studies, about 48% of the participants had a wrong concept before the intervention that reducing dietary intake of sugar and food additives was one modality of reducing ADHD symptoms [19]. However, no scientific evidence supports that sugar plays a role in the development of ADHD or in managing the symptoms [20].

The results of the current study have indicated that the educational intervention had a substantial influence on teachers' knowledge regarding ADHD, confirming earlier studies that have shown the value of short-term programs in raising teachers' understanding of ADHD [21]. The findings showed a statistically significant disparity between the study group's pre- and post-intervention scores on the KADDS relating to general symptoms and treatment, with improved knowledge scores in all domains immediately after the intervention. This result agreed with the previous Saudi study that reported the effectiveness of a two-day workshop compared to earlier interventional studies where training had to occur across several days [12,22]. A recent study also demonstrated that the effectiveness of educational interventions proved important in increasing knowledge in general in society, regardless of the training program method [23]. Therefore, the effectiveness of the educational programs can be regulated according to the time and resources currently available and the number of trainers affordable in each school with acceptable learning outcomes.

This poor pre-intervention knowledge among teachers was associated with gender and the previous attendance of training workshops; this result is consistent with the last Saudi studies that confirmed that the previous training positively influences knowledge [18,24]. However, the poor knowledge score declined post intervention and was significantly associated with age, residence, years of experience, and the attendance of ADHD training workshops.

The current study highlights teacher training and examines the effectiveness of available short-term educational intervention resources for ADHD. However, several limitations must be taken into account when evaluating our findings. First, because of the limited duration of the intervention, it could not studied whether the long-term benefits could be influenced by the teacher's attention span and general mood over such a short period; it also reduces reassurance beyond the intervention's expanded learning outcome. Second, because the questionnaire was self-reported, participants may have overestimated their understanding of ADHD, which may be one reason for the wrong rate of adequate knowledge. Moreover, we grouped the incorrect and 'don't know' answers together and assigned them the same score, making it challenging to distinguish teachers' intention of not answering from poor knowledge. Lastly, the relatively small sample size and the study being conducted in a single city and only in public schools limit the generalizability of the results, so more extensive research is warranted in the future to validate the results. 

## Conclusions

Teachers play a crucial role in helping manage ADHD-affected children as they usually spend significant time with children and can promptly observe the behavioral changes in ADHD children. In this study, the teachers' knowledge regarding ADHD showed considerable improvement after implementing an educational intervention. Although most previous research in Saudi Arabia has shown that there is still insufficient knowledge, wrong perception, and lack of positive attitude surrounding ADHD among teachers, such short-term educational intervention can participate in improving their knowledge in cost-effective ways. The teacher's knowledge and motivation for dealing with children with ADHD would be enhanced by attending training workshops and integrating the particular education course into the instructors' educational curriculum. 

## Appendices

**Table 6:** The KADDS demonstrates the ADHD domains General criteria domain: 1,4,6,13,17,19,22,24,27,28,29,30,31,32,33; Symptoms and diagnosis domain: 3, 5,7,9,11,14,16,21,26; Treatment domain: 2,8,10,12,15,18,20,23,25,34,35,36 KADDS: Knowledge of Attention Deficit Disorders Scale; ADHD: attention deficit hyperactivity disorder

	#	Item	incorrect	Don’t know	correct
1		Most estimates suggest that ADHD occurs in approximately 15% of school-age children.			
2		Current research suggests that ADHD is largely the result of ineffective parenting.			
3		ADHD children are frequently distracted by external stimuli.			
4		ADHD children are typically more compliant with their fathers than with their mothers.			
5		To be diagnosed with ADHD, the child’s Symptoms must have been present before age seven.			
6		ADHD is more common in the first-degree biological relatives (e.g., mother and father) of children with ADHD than in the general population.			
7		One symptom of ADHD is that they have been physically cruel to others.			
8		Antidepressant drugs have been effective in reducing symptoms for many ADHD children.			
9		ADHD children often fidget or squirm in their seats			
10		Parent and teacher training in managing an ADHD child is generally effective when combined with medication treatment.			
11		It is common for ADHD children to have an inflated sense of self-esteemed or grandiosity.			
12		When treatment of an ADHD child is terminated, It is rare for the child’s symptoms to return.			
13		It is possible for an adult to be diagnosed with ADHD.			
14		ADHD children often have a history of stealing and destroying other people’s things.			
15		Side effects of stimulant drugs used to treat ADHD may include mild insomnia and appetite reduction.			
16		Current wisdom about ADHD suggests two clusters of symptoms: one of inattention and another consisting of hyperactivity-impulsivity.			
17		Symptoms of depression are found more frequently in ADHD children than in non-ADHD children.			
18		Individual psychotherapy is usually sufficient for the treatment of most ADHD children.			
19		Most ADHD children outgrow their symptoms by puberty and subsequently usually function in adulthood.			
20		in severe cases of ADHD, medication is often used before other behavior modification techniques are attempted.			
21		In order to be diagnosed with ADHD, a child must exhibit relevant symptoms in two or more settings (e.g., at home,, and school).			
22		If an ADHD child can demonstrate sustained attention to video games or TV for over an hour, that child can also sustain attention for at least an hour of class or homework.			
23		Reducing dietary intake of sugar or food additives is generally effective in reducing symptoms of ADHD.			
24		a diagnosis of ADHD by itself makes a child eligible for placement in special education.			
25		Stimulant drugs are the most common drug used to treat children with ADHD.			
26		ADHD children often have difficulty organizing tasks and activities.			
27		ADHD children generally experience more problems in novel situations than in familiar situations.			
28		There are specific physical features that can be identified by medical doctors (eg, pediatricians) in making a definitive diagnosis of ADHD.			
29		In school-age children, the prevalence of ADHD in males and females is equivalent.			
30		In every young child (less than four years old), The problem behaviors of ADHD children (e.g., hyperactivity, inattention) are distinctly different from age-appropriate behaviors of non-ADHD children.			
31		Children with ADHD are more distinguishable from normal children in a classroom setting than in free-play situations.			
32		The majority of ADHD children evident some degree of poor school performance in the elementary school years.			
33		Symptoms of ADHD are often seen in non-ADHD children who come from inadequate and chaotic home environments.			
34		Behavioral /psychological interventions for children with ADHD focus primarily on the child’s problem with inattention.			
35		Electroconvulsive therapy(i.e., shock treatment) is an effective treatment for severe cases of ADHD.			
36		Treatments for ADHD, which focus primarily on punishment, are the most effective in reducing the symptoms of ADHD.			
